# Crochetage Sign on Electrocardiography as a Diagnostic Marker of Patent Foramen Ovale

**DOI:** 10.1016/j.jaccas.2026.108235

**Published:** 2026-07-29

**Authors:** Suraj S. Patel, Matthew D. Atienza, Mark B. Hellerman, David T. Zhang

**Affiliations:** a Renaissance School Medicine, Stony Brook University, Stony Brook, New York, USA; b Division of Cardiology, Department of Medicine, Stony Brook Medicine, Stony Brook, New York, USA

**Keywords:** crochetage sign, patent foramen ovale, PFO

## Abstract

**Introduction:**

A young man was hospitalized for evaluation of acute neurologic symptoms. Initial diagnostic testing was unrevealing; however, a 12-lead electrocardiogram demonstrated notching at the apex of the R wave in the inferior leads, consistent with the crochetage sign.

**Case Presentation:**

Further cardiac evaluation was pursued. Transthoracic echocardiography identified an atrial septal aneurysm with evidence of interatrial shunting on agitated saline contrast study, consistent with a patent foramen ovale.

**Discussion:**

This case highlights the diagnostic value of careful electrocardiographic interpretation and demonstrates how subtle electrocardiogram findings may strongly suggest underlying structural heart disease.

## Graphical Abstract

**Figure:**
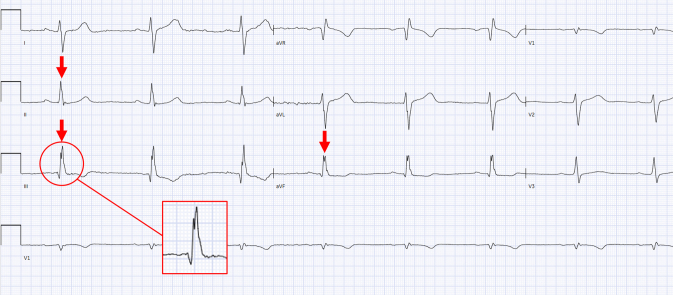

## History of Presentation

A 21-year-old man presented with intermittent paresthesia. Past medical history was notable for Lyme disease. He was taking no medications. Presenting vital signs were normal. Initial physical examination, laboratory testing, and imaging were unremarkable. A screening electrocardiogram (ECG) demonstrated sinus rhythm with characteristic notching at the apex of the R wave in the inferior limb leads (Figure 1). Given the patient's neurologic symptoms, a cardioembolic etiology was considered.

**Take-Home Messages**

• The crochetage sign is a specific electrocardiographic marker of interatrial septal abnormalities.
• Subtle ECG findings can identify clinically significant structural heart disease even in patients with competing diagnostic considerations.

**Figure 1:**
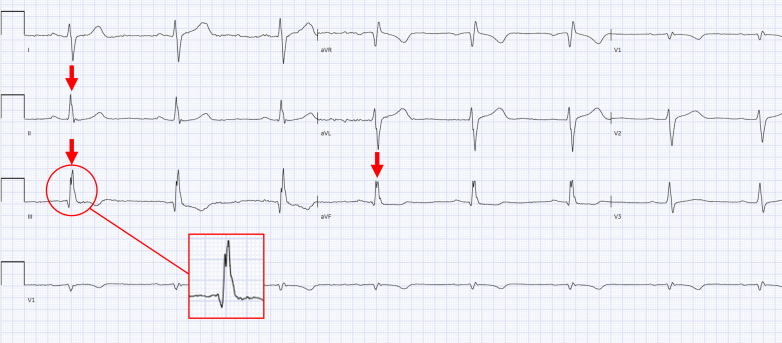
12-Lead Electrocardiogram Notching at the apex of the R-wave in the inferior limb leads (arrows) is consistent with the crochetage sign.

## Question

Which of the following best describes the electrocardiographic finding and its most likely clinical implication?

A. Incomplete right bundle branch block reflecting right ventricular pressure overload
B. Inferior lead R-wave notching suggesting an interatrial septal abnormality
C. Right axis deviation with secondary ST-T wave changes consistent with pulmonary hypertension
D. Fragmented QRS complexes indicating prior myocardial scarring

## Discussion and Rationale

Choice A is incorrect. Incomplete right bundle branch block may be seen in patients with atrial septal defects due to right ventricular volume overload; however, it does not account for focal notching at the apex of the R wave in the inferior leads.

Choice B is correct. The ECG demonstrates notching at the apex of the R-wave in the inferior limb leads, a pattern known as the crochetage sign. This electrocardiographic finding has been described as a specific marker of interatrial septal abnormalities, particularly secundum atrial septal defect, and has been reported in association with patent foramen ovale (PFO).^1^ When present across multiple inferior leads, the crochetage sign exhibits high specificity for an underlying interatrial shunt and may occur independently of right bundle branch block patterns.^1^ In this case, recognition of this characteristic ECG pattern prompted targeted structural evaluation, with transthoracic echocardiography demonstrating an atrial septal aneurysm and agitated saline contrast study revealing an interatrial shunt consistent with PFO.

Choice C is incorrect. Right axis deviation with secondary ST-T wave changes may be associated with pulmonary hypertension and chronic right ventricular pressure overload. In this ECG, there is no marked axis deviation or repolarization abnormality to suggest pulmonary hypertension.

Choice D is incorrect. Fragmented QRS complexes are typically characterized by multiple small deflections within the QRS complex across contiguous leads and are associated with myocardial scarring or fibrosis. The isolated, consistent notching at the apex of the R wave confined to the inferior leads in this ECG is not characteristic of fragmented QRS.

The electrophysiologic basis of the crochetage sign is incompletely defined, but is thought to reflect altered atrial activation related to interatrial shunting and septal distortion. Prior studies have demonstrated correlations between this ECG finding and shunt magnitude, as well as attenuation following defect closure, supporting its relationship to shunt-related hemodynamics.^1^ Although most commonly described in atrial septal defect, the crochetage sign has also been reported in patients with PFO, particularly in the setting of ischemic stroke, where it may serve as a noninvasive clue to paradoxical embolic risk.^2^ Lyme disease is a multisystem infection that may involve both the nervous system and the cardiac conduction system, most commonly manifesting with atrioventricular block due to Lyme carditis.^3^ Although the history of Lyme disease in this patient may explain his neurologic symptoms, the ECG findings in this case instead point toward an underlying interatrial shunt. Notably, the ECG does not demonstrate a classic right bundle branch block pattern, as expected terminal S waves in the lateral leads are absent, emphasizing that the crochetage sign may occur independently of conventional conduction abnormalities. This case highlights the importance of careful electrocardiographic interpretation, as subtle ECG findings alone may strongly suggest underlying congenital cardiac abnormalities and guide focused diagnostic evaluation.

## Funding Support and Author Disclosures

The authors have reported that they have no relationships relevant to the contents of this paper to disclose.
